# Annotations

**Published:** 1899-01-07

**Authors:** 


### Annotations.

### Unmarried Women and Maternity Hospitals.

The recent death of Miss Yates, in connection with which Robert Wark was convicted of murder at the last Liverpool Assizes, has directed public attention to the desirability of establishing maternity hospitals where (as in Queen Charlotte's Hospital) unmarried women may be admitted for a first confinement. A special meeting of the subscribers to the Liverpool Ladies' Charity and Lying-In Hospital was held last week to consider the subject, as, according to the rules of that institution, its benefits are conferred only on married women. At this meeting it was moved by Mrs. Henry Tate: "That single women in exceptional circumstances, who, after careful investigation by the Ladies' Committee, are found to be deserving objects of charity, shall be eligible for admission into the hospital for their first confinement." Lady Derby had sent a letter, in which she expressed deep sympathy with the proposal, and promised aid in every possible way. The resolution was carried by 45 votes to 31, and it was decided to alter the rules accordingly. We need hardly say that charity covereth so many sins that it may well cast its cloak over some of the cases referred to. At the present time public lying-in hospitals are few and far between, and no great harm to public morality will be done by such a relaxation of rule as is here suggested. At the same time, we would point out that the question is a large one, and that, if admission to such institutions should ever become as easy as admission to some other special hospitals is at the present time, very serious questions might arise in regard to their effect upon some of the very foundations of our social system.

### Death in the Bath.

An unfortunate accident—for we will call it an accident in deference to the feelings of a probationer nurse who was overwrought at the time, and who, we are sure, has suffered much unhappiness from the unfortunate occurrence—has taken place at the Radcliffe Infirmary, Oxford, which renders it imperative that we should urge not only upon nurses, but more especially upon those who have the management of hospitals, the necessity of bearing in mind and of enforcing the standing rule that a young child should not be left alone in a bath. It is a simple matter, but its neglect may mean death, as it did at Oxford, and as it has done many a time before. A probationer in her second year is put in night charge of the children's ward; she goes on duty at nine o'clock in the evening and remains until eight next morning. She is visited every two hours by the night nurse on her rounds, but otherwise is alone with her thirteen little patients. All the children that are well enough to have a bath have to be bathed by her, and this has to be done before seven o'clock in the morning. On this particular morning nine baths have to be given. Some time before five o'clock the process begins. A little boy, two and a-half years old, is roused and taken to the bath-room. The little chap is popped into the bath, where he is left standing while the nurse carries another child who had just been bathed into the ward to finish drying her. Then she makes her bed and turns the boy's mattress ready for his return, and, when she goes to fetch him, finds him dead—laid in the water "quite doubled up, his face on his feet." Nothing could be more simple or more tragic. Standing in a slippery bath, his feet slip from under him; at once his mouth is under water. There is no cry, for at the first gasp his lungs are filled with water, and he is drowned, and all because of the lack of a simple rule that a child in a bath should never be left, never under any circumstances. The nurse, being asked, "Were any instructions ever given you about leaving the children alone in the bath-room?" answered, "Never." The matron, on examination, is reported to have said "she considered it an error of judgment to leave a child in the bath alone." But ought it not rather to have been the breaking of a positive rule? In answer to a further question, "witness did not know whether the nurse was ever told not to leave a child alone in the bath." And if deep baths continue to be used for infants, and if the wholesome rule of "one at a time" is not enforced, such fatalities as this will continue to recur.

### The Meanness of Well-to-do Man.

While Dr. Whiteside Hime, of Bradford, is entertaining the good people of the West Riding of Yorkshire with examples of the meanness of well-to-do people in his part of the world in applying to charity hospitals for medical relief, and thus appropriating to their own use money which has been subscribed by a confiding public for the sick poor, we receive from the Antipodes a long string of examples of the same sort of meanness which leads well-to-do Australians to cheat the medical profession by joining Friendly Societies and obtaining the services of the club doctors for the threepence a week which in that country seems to be the standard club fee. According to the report of a recent Inter-Colonial Medical Conference, published in the *Inter-Colonial Medical Journal of Australia* one of these 3d.-a-week patients lives in a mansion worth £5,000, and his wife owns and runs a racehorse; another is a retired Civil servant owning a row of cottages who, while taking his ordinary attendance from his club doctor at 3d. a week, does not hesitate to pay two guineas as a consulting fee when he requires further advice. One of the medical men making this report has attended an ex-mayor with many thousands. He paid him about two hundred visits and consultations during three years, and got nothing beyond his 3d. a week. Another has attended a bank manager, two brewers, several well-to-do storekeepers, and a good many farmers. One doctor says he attended a man worth from £20,000 to £30,000, who lived in a castle in an eastern suburb; while another had a patient whose will was proved at £22,000, yet he had attended him and his family for 13s. per annum. There are pages of these examples, and the universal testimony is, that while the *bona fide* working man, the proper club patient, is comparatively considerate, these well-to-do parasites on the club system are troublesome and exacting to a degree. One doctor says they "expect more, are less thankful, and always have an idea they are not getting enough for their money," while he plaintively adds: "The wife generally keeps you waiting a quarter of an hour in the drawing-room while she 'does' her hair." The "drawing room" of a patient at 3d. a week! Alas! for the meanness of well-to-do man!

